# Measurement of the Rheological Properties of High Performance Concrete: State of the Art Report

**DOI:** 10.6028/jres.104.028

**Published:** 1999-10-01

**Authors:** Chiara F. Ferraris

**Affiliations:** National Institute of Standards and Technology, Gaithersburg, MD 20899-0001

**Keywords:** building technology, concrete, flow properties, mortar, rheology, rheological measurements, rheological models, test methods, suspension, workability

## Abstract

The rheological or flow properties of concrete in general and of high performance concrete (HPC) in particular, are important because many factors such as ease of placement, consolidation, durability, and strength depend on the flow properties. Concrete that is not properly consolidated may have defects, such as honeycombs, air voids, and aggregate segregation. Such an important performance attribute has triggered the design of numerous test methods. Generally, the flow behavior of concrete approximates that of a Bingham fluid. Therefore, at least two parameters, yield stress and viscosity, are necessary to characterize the flow. Nevertheless, most methods measure only one parameter. Predictions of the flow properties of concrete from its composition or from the properties of its components are not easy. No general model exists, although some attempts have been made. This paper gives an overview of the flow properties of a fluid or a suspension, followed by a critical review of the most commonly used concrete rheology tests. Particular attention is given to tests that could be used for HPC. Tentative definitions of terms such as workability, consistency, and rheological parameters are provided. An overview of the most promising tests and models for cement paste is given.

## 1. Introduction

The rheological (flow) properties of concrete are important for the construction industry because concrete is usually put into place in its plastic form. This importance can be attested to by the large body of literature existing on concrete rheology [1,2,3,4]. Unfortunately, due to the complex composition of the material, no definite method for predicting the flow of concrete from its components exists. Even measurements of the rheological parameters are not easily performed due to the large range of particle sizes found in concrete (from 1 mm cement grains to 10 mm coarse aggregates or even larger (100 mm) as found in a dam). Therefore, the flow of a given concrete is usually measured using one of the many standard tests^1^ available that only partially measure the intrinsic flow properties of the material. Flow tests are of limited value unless they measure the intrinsic rheological properties of concrete. A better understanding of the flow properties of concrete is needed to be able to predict the flow of concrete from the properties of the components.

The purpose of this paper is to assess the state of the art in measurements of flow properties of concrete. A critical review of the tests available is given with special emphasis given to tests for high performance concrete (HPC). Definitions of terms commonly used in the field and their link to material properties are provided.

## 2. Theoretical Background

### 2.1 Fluid And Suspension Rheology

Concrete and mortar are composite materials, with aggregates, cement, and water as the main components. Concrete is really a concentrated suspension of solid particles (aggregates) in a viscous liquid (cement paste). Cement paste is not a homogeneous fluid and is itself composed of particles (cement grains) in a liquid (water).

**Fig. 1:**
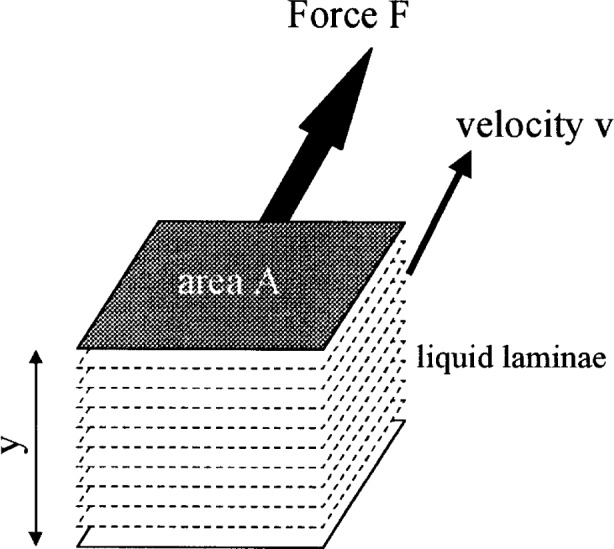
Newton’s equation of viscous flow [4].

Because concrete, on a macroscopic scale, flows as a liquid, equation (1) is applicable. If a shear force is applied to a liquid as shown in Fig. 1, a velocity gradient is induced in the liquid. The proportionality factor between the force and the gradient is called the viscosity. The velocity gradient is equal to the shear rate $\dot{\gamma }$. A liquid that obeys this equation is called Newtonian [1].

$$F/A=\tau =\eta \;\dot{\gamma }$$ (1)

where

*η* = viscosity
$\dot{\gamma }$ = shear rate = d*v*/d*y* (see Fig. 1)
*τ* = shear stress = *F*/*A*
*F* = shear force
*A* = area of plane parallel to force.

Most of the equations used for concentrated suspensions, such as concrete, try to relate the suspension concentration to the viscosity or the shear stress to the shear rate, thus assuming that there is only one value for the viscosity of the whole system. Table 1 and Table 2 give the most commonly used equations in the two approaches. Equations from Table 1 are used to describe the flow of cement paste [5], but they are not applicable to concrete due to the complexity of the suspension (aggregates in a suspension (cement paste)). Table 2 gives equations commonly used for concrete.

**Fig. 2:**
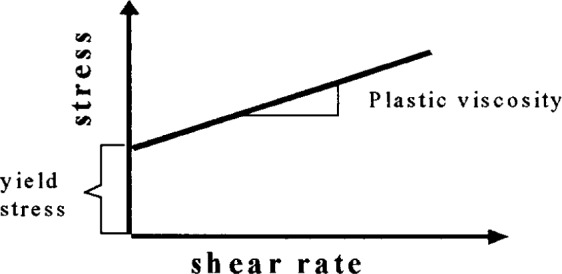
Bingham’s equation for a fluid.

It should be noted that quite a few of the equations described in Table 2 incorporates a second factor, the yield stress. The physical interpretation of this factor is that the yield stress is the stress needed to be applied to a material to initiate flow. For a liquid, the yield stress equal to the intersection point on the stress axis and the plastic viscosity is the slope of the shear stress-shear rate plot (see Fig. 2). A liquid that follows this linear curve is called a Bingham liquid.

**Fig. 3:**
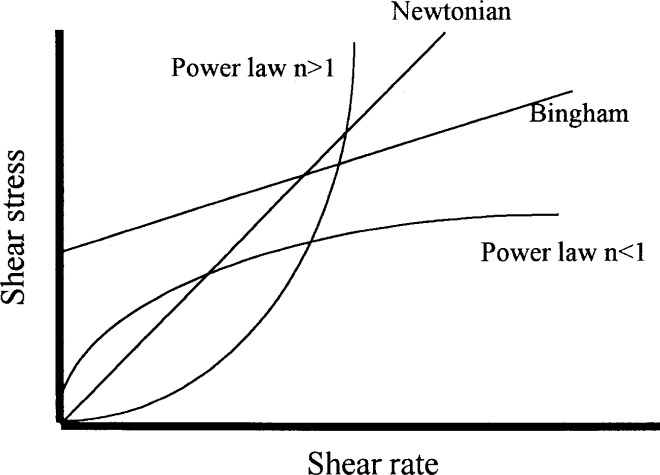
Summary of shapes of shear stress-shear rate curves [1].

Figure 3 shows some of the idealized types of curves that can be obtained when shear stress is plotted against shear rate. All the curves depicted can be described by one of the equations of Table 2. Liquids following the power law are also called pseudo-plastic fluids.

Atzeni et al. [7] have compared the various equations and proposed a modification of the Eyring equation as the best fit for concentrated suspensions such as cement paste. Unfortunately, the parameters of the Eyring equations are not physical values, but fit variables. Therefore, these parameters cannot be measured independently or modeled, but are calculated by a best-fit routine.

The main conclusion that can be deduced from studying the proposed equations is that all (with the exclusion of the Newtonian liquid) use at least two parameters to describe the flow. In the case of a concentrated suspension such as concrete, it has been shown [4,5] that a yield stress exists. The equations that have a physical basis include at least two parameters, with one being the yield stress, are the Herschel-Bulkley and Bingham equations. The Herschel-Bulkley equation contains three parameters, one of which, *n*, does not represent a physical entity. It has been shown [10] that in certain concretes, such as self-consolidating concretes, this is the equation that best describes their behavior. Nevertheless, the most commonly used equation today is the Bingham equation, because the parameters used are factors that can be measured independently (Fig. 2) and because the flow of real concrete seems to follow this equation fairly well [4] in most cases.

### 2.2 Concrete Rheology

In the construction field, terms like workability, flowability, and cohesion are used, sometimes interchangeably, to describe the behavior of concrete under flow. The definitions of these terms are very subjective. Table 3 [14] lists some of the major definitions of workability given by professional societies. Tattersall’s [4] interpretation of workability is “the ability of concrete to flow in a mold or formwork, perhaps through congested reinforcement, the ability to be compacted to a minimum volume, perhaps the ability to perform satisfactorily in some transporting operation or forming process, and maybe other requirements as well”. Kosmatka et al. [11] mention the following three terms while referring to concrete rheology: workability, consistency and plasticity. The definitions given are:

- “Workability is a measure of how easy or difficult it is to place, consolidate, and finish concrete”
- “Consistency is the ability of freshly mixed concrete to flow”
- “Plasticity determines concrete’s ease of molding”.

It is clear that the definitions are descriptive and no agreement can be found. In the field, the situation is often worse because these terms are used differently by the various persons involved. From the previous list, all the terms used are defined according to the feelings of the person and are not based from the physical behavior of the material. Richtie [12] attempted to define the flow of concrete by linking it to various effects such as bleeding, sedimentation, and density. He distinguishes three properties: stability, compactibility, and mobility. The stability is linked to bleeding and segregation. The compactibility is equivalent to density, while mobility is linked to internal friction angle, bonding force, and viscosity. These descriptions, at least, link commonly used words with physical factors that can be measured. However, we believe that this is not enough. All these terms should be discarded in favor of physically measurable parameters. For instance, we could say that a concrete has a higher viscosity, instead of referring to a lower workability. Tattersall [4] summarizes very clearly the concrete workability terminology by classifying it into three classes: qualitative, quantitative empirical, and quantitative fundamental. The following items fall in the three classes.

- Class I: qualitative
  Workability, flowability, compactibility, stability, finishability, pumpability, consistency, etc. To be used only in a general descriptive way without any attempt to quantify.
- Class II: quantitative empirical
  Slump, compacting factor, Ve-be, etc. To be used as a simple quantitative statement of behavior in a particular set of circumstances.
- Class III: quantitative fundamental
  Viscosity, yield stress, etc. To be used in conformity with the British Standard Glossary [13].

As stated in Sec. 2.2, the properties that could be used to describe the concrete flow are the yield stress and the viscosity. Any test that describes the flow behavior of concrete should at least measure these two properties. Unfortunately, most existing tests measure only one factor, either related to the yield stress or to the viscosity. Descriptions of these tests are given in Sec. 3.1. Tests measuring both parameters exist but are neither cheap nor easy to carry out, so they are not widely used. Section 3.2 describes these tests.

Aside from measuring the flow of concrete, rheology is concerned with the prediction of the flow from the properties of the components (i.e., cement paste, mortar) or from the mix design (i.e., w/c ratio, aggregate content, type of cement and admixture dosage). No attempt to develop a prediction model has yet been successful. One difficulty comes from the fact that the size range of the particles is very wide (micrometers to tens of millimeters). Also, the factors influencing the flow properties of concrete are more than the factors influencing the rheology of the parts (cement paste and aggregates). There is no linear relationship between the rheological parameters of cement paste and those of concrete. The main reason being the gap between the aggregates which varies with the concrete cement paste volume content. Ferraris et al. [15] showed that cement paste has a different rheological behavior depending on the gap between the plates of a rheometer that simulate the distance between the aggregates. The distance between the aggregate depends on the cement paste volume content. Also, the rheological behavior of a material depends on the conditions of the experiment such as shear rates, temperature, mixing energy. Therefore, it is important that the cement paste be measured in the same conditions that it will experience in concrete. This approach was followed by Yang et al. [16] to determine the influence of mixing methods on the flow properties of cement paste. Martys [17] is currently attempting to develop a simulation of the flow of concrete using a computerized model with the mix design and the cement paste rheology measured under the same conditions as in concrete as input variables. De Larrard [18] developed a model based on optimization of mixture design, linking maximum close packing with concrete properties. Further description of these models is beyond the scope of this paper.

It should be kept in mind that all the models or methodologies given here assume that the concrete is formed of particles but no interparticle forces are directly considered. The only reference to particle interaction is the acknowledgment that all properties are time-dependent, implying that phenomena, such as flocculation of the cement particles and hydration, are continually taking place.

**Fig. 4:**
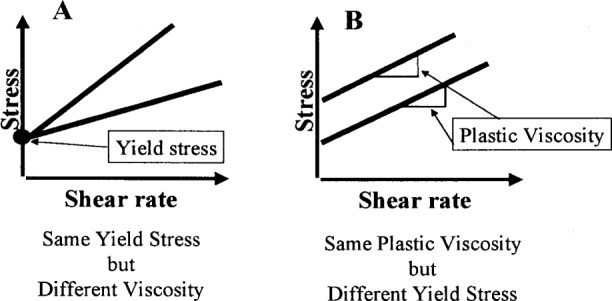
Concrete rheology.

In summary, concrete is a suspension, including particles that may range from less than 1 mm to over 10 mm. The flow properties of such a suspension can often be described approximately using a Bingham model, defined by two factors, plastic viscosity and yield stress. Most of the widely used tests are unsatisfactory in that they measure only one parameter, which does not fully characterize the concrete rheology. Figure 4 shows how two concretes could have one identical parameter and a very different second parameter. These concretes may be very different in their flow behaviors. Therefore, it is important to use a test that will describe the concrete flow, by measuring (at least) both factors.

### 2.3 High Performance Concrete

High performance concrete (HPC) is defined by ACI [19] as follows: “HPC is a concrete meeting special combinations of performance and uniformity requirements that cannot always be achieved routinely using conventional constituents and normal mixing”. The rheological property of HPC should be: “HPC places and compacts easier” [20]. In a detailed characterization of HPC, given by Goodspeed et al. [21], the reference to rheology is: “Ease of placement and consolidation without affecting strength”.

To achieve this property special precautions need to be taken. According to Malier [22], a more workable HPC can be obtained in two ways: either by reducing the flocculation of cement grains or by widening the range of grain sizes. Examining Malier’s method, it is apparent that the first approach relates uniquely to the cement paste, while the second approach relates to the aggregate’s size distribution as well as the influence of fillers. The aggregate’s size distribution is at the base of the computerized calculation of concrete mixture design developed by Shilstone [23].

Aitcin [24] has raised several questions on the production of a more workable HPC:

- “How to evaluate simply rheological performance of portland cement and its compatibility with given superplasticizers?
- How to evaluate simply in the laboratory and in the field, the workability of a concrete having a very low water/cement ratio by means other than the slump test?
- How is diminished the rheological performance of a given portland cement in the domain of low water/cement ratio?
- How to optimize the use of supplementary cementitious materials when making low water/cement ratio concrete?”

Today, the workability of HPC is evaluated using the same tests as used for normal concrete. However, the specific characteristics of HPC hinder the correct interpretation of current tests. This situation is demonstrated when the yield stress, as measured by a slump cone, is in the range desired but the viscosity (not measured in a slump cone test) may be so high that the mix is labeled “sticky” and is difficult to place in the molds even with vibration.

Therefore, new tests are being designed specifically for HPC, as will be described in Sec. 3.2.3.

## 3. Test Methods

Test methods for flow properties of concrete can be divided into two groups in regard to whether the output of the experiment gives one or two parameters. As was discussed in Sec. 2.2, to correctly define the rheology of concrete both the yield stress and the viscosity need to be measured.

### 3.1 One-Factor Tests

Most currently used tests measure only one rheological value or factor. The relationship between the factor measured and either of the two fundamental rheological parameters is not obvious. In most cases, the fundamental parameter cannot be calculated from the factor measured, but can only be assumed to be related. The tests that are discussed here are (all references will be given in the sections where the tests are discussed):

1. Slump
2. Penetrating rod: Kelly ball, Vicat, Wigmore test
3. K-slump test
4. Ve-Be time or remolding test (Powers apparatus)
5. LCL apparatus
6. Vibration testing apparatus or settling curve
7. Flow cone
8. Turning tube viscometer
9. Filling ability
10. Orimet apparatus.

Tests 1 through 3 are related to the yield stress because they measure the ability of concrete to start flowing. Tests 4 to 10 are related to the viscosity because they measure the ability of concrete to flow after the stress exceeds the yield stress. The stress applied is either by vibration (tests 4–6) or by gravity (tests 7–10).

#### 3.1.1 Slump Test

**Fig. 5:**
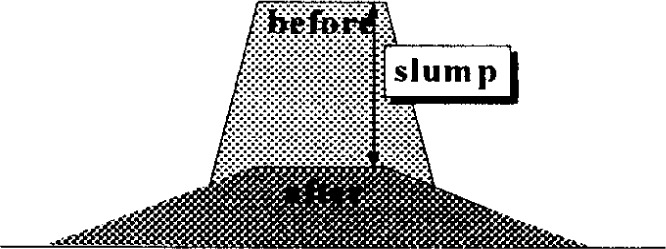
Schematic view of the slump test.

**Fig. 6:**
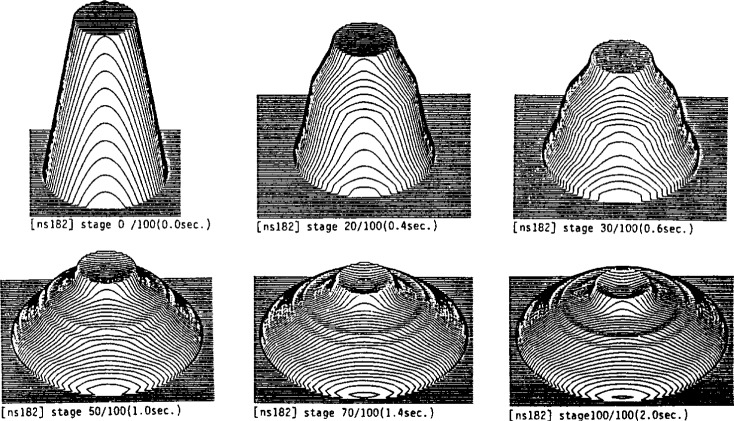
Finite element simulation of a slump cone test [26] (reprinted with permission from the Japan Concrete Institute).

A truncated metal cone, open at both ends and sitting on a horizontal surface, is filled with concrete, and lifted quickly. The slump of the concrete is measured as shown in Fig. 5. This measurement is widely used due to its simplicity. In this test, the stress is composed of the weight of the concrete per unit area. The concrete will slump or move only if the yield stress is exceeded and will stop when the stress (or weight of the concrete/area) is below the yield stress. Therefore, the slump test is related to the yield stress [25]. Some researchers have tried to simulate the slump test [26] using the finite element method. Assuming that concrete follows the Bingham equation, they were able to produce pictures of the concrete slump versus time (Fig. 6), but no prediction from the concrete composition was possible because no material properties for the components (cement paste, aggregates) were used.

The variability in the slump measurements is attributed mainly to the operator and to variations in mixture proportions. This test is a useful quality control tool because it can help detect changes in the composition of concrete delivered, e.g., changes in the amount of mixing water. This test is a standard in the United States (ASTM C143) [27] and is used in other countries as well.

**Fig. 7:**
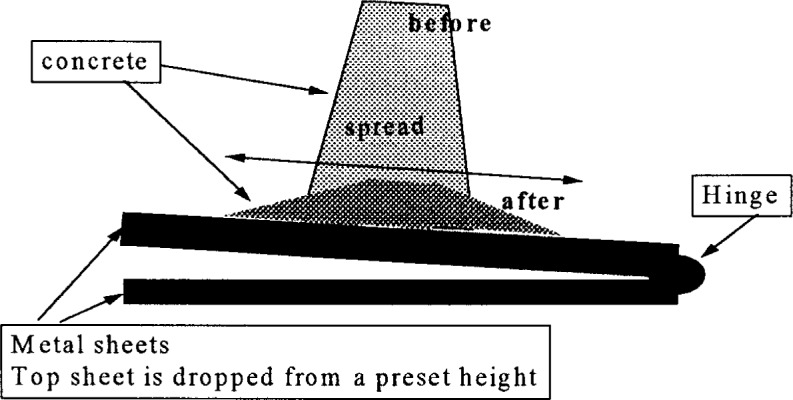
Slump cone according to DIN 1045 [28].

A modification of the slump test, used for concretes with very high slump (as high as 305 mm (12 in) minus the coarser aggregate diameter), is to measure the spread instead of the height drop. This measurement is rarely reported and is not a standard. A second modification of the slump cone (see Fig. 7) is the test used in Germany (DIN 1045) [28]. The slump cone is placed on a special metal sheet. After the cone is lifted, the metal sheet is lifted and dropped a predetermined number of times. The spread of the concrete is measured. This version of the slump cone test is related to the viscosity and not to the yield stress because dropping the metal sheet subjects the concrete to a stress that is greater than the yield stress. Therefore, the measurement is related to the flow of concrete when the yield stress is exceeded. If the concrete does not slump or spread, than this measurement is not useful because the yield stress was not exceeded and the concrete did not flow. This statement can be applied also to the measurement of the standard slump of the concrete.

Recently, the slump cone test procedure was modified to allow the estimation of both the yield stress and the viscosity [29,30]. As the modified slump cone test is classified as a test for two parameters, it will be described in Sec. 3.2.

#### 3.1.2 Penetrating Rod: Kelly Ball, Vicat, and Wigmore Tests

**Fig. 8:**
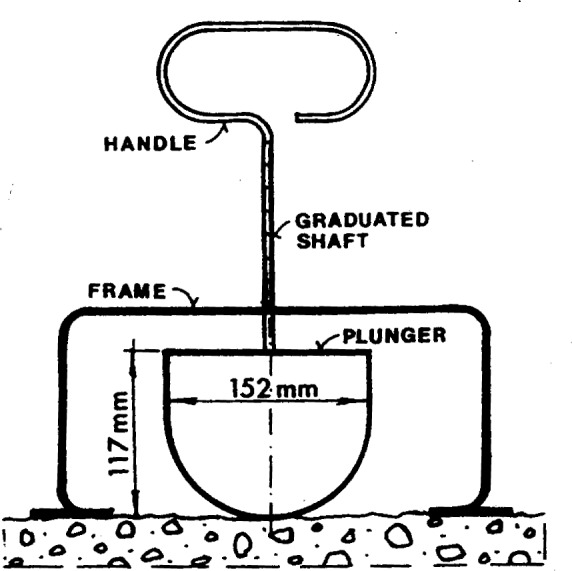
Penetration tests: Kelly Ball [33]. (Reprinted with permission from the Annual Book of ASTM Standards, copyright American Society for Testing and Materials, 100 Barr Harbor Drive, West Conshohocken, PA 19428.)

**Fig. 9:**
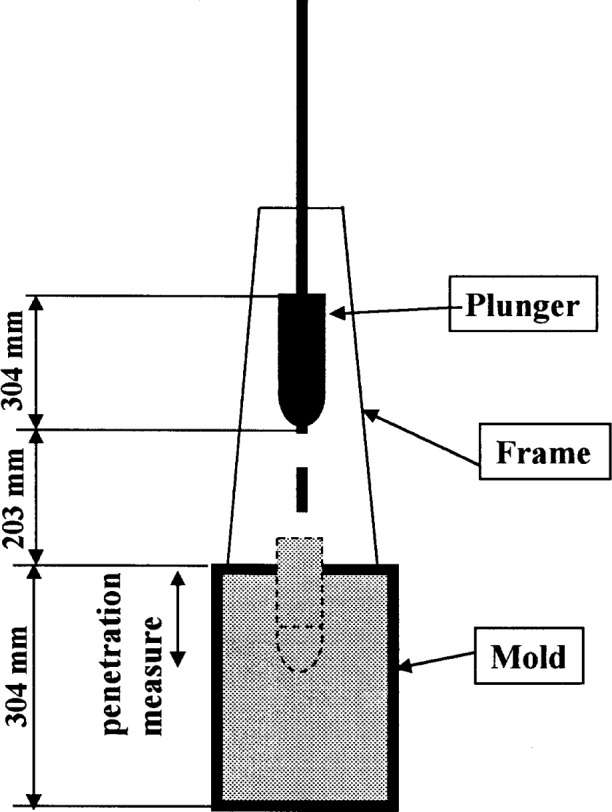
Penetration tests: German penetration apparatus [28].

The principle of these tests is that the depth of penetration of an object will depend on the yield stress of the concrete. The mass or the force applied on the penetrating object will measure the yield stress of the concrete. Usually, the mass or the force is pre-established, i.e., it does not vary depending on the sample. Therefore, these tests really measure whether the applied stress is higher or lower than the yield stress of the concrete. Similar to the slump test, these tests are useful mainly on work sites as quality control tools to determine if the composition (mainly the water content) has been changed. These tests are also frequently used to determine the setting time of concrete. Figures 8 and 9 show two of the most known configurations. Other test descriptions can be found in ASTM C 403 [31] or, for the Vicat needle, in ASTM C 953 [32].

It should be noted that the Kelly Ball described in ASTM C360 [33] was up for reapproval in 1998. It failed to pass, due to lack of use, therefore it will likely be withdrawn as a ASTM standard.

#### 3.1.3 Turning Tube Viscometer

**Fig. 10:**
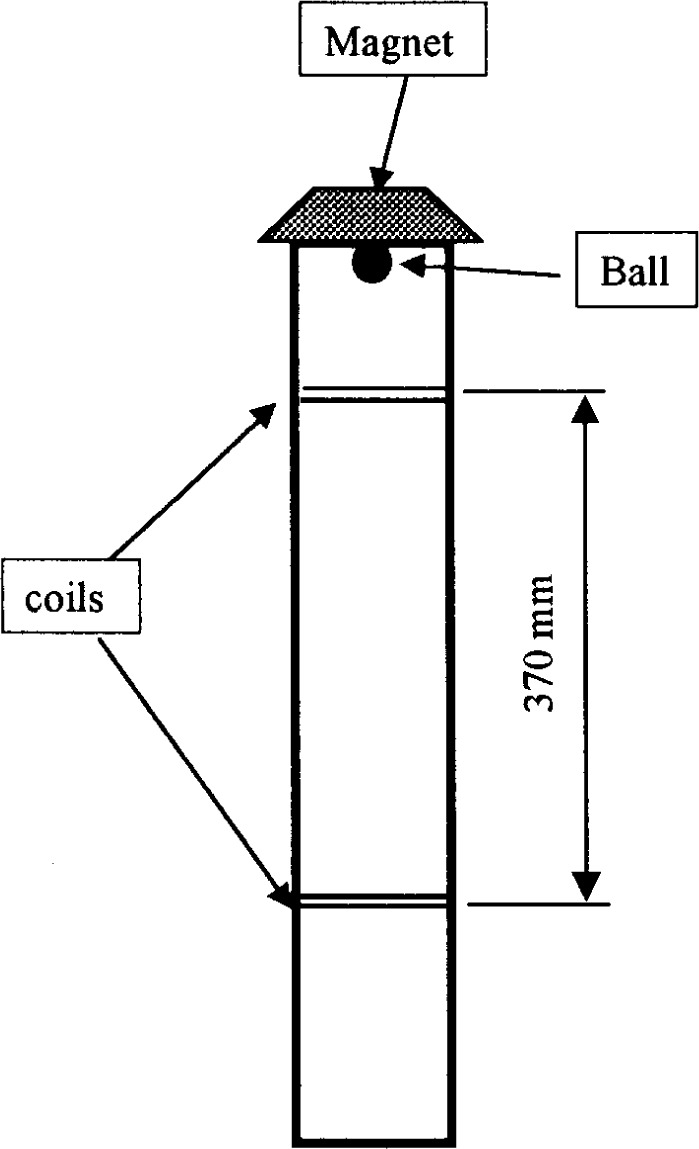
Schematic drawing of the turning-tube viscometer [34].

The turning tube viscometer consists of a tube (60 mm in diameter and 800 mm long) that can be filled with the material to be measured [34]. A ball is then dropped in the fluid and its velocity measured between two points 370 mm apart [the two inductance coils will detect the ball passing (Fig. 10)]. The ball sizes are 12.7 mm, 15.9 mm, and 24.9 mm. Using the Stokes equation, the viscosity is calculated. This instrument has been used to measure the viscosity of cement paste. It is not recommended for concrete, because the diameter of the ball should be significantly larger than that of the aggregates. Otherwise, the concrete cannot be considered to be a uniform medium in which the ball is freely falling. Also, the diameter of the tube needs to be large enough to insure that the coarser aggregates do not interlock and stop the ball’s descent.

#### 3.1.4 K-Slump Test [35,36]

This test was widely used [35] before it became an ASTM standard in 1997 [36]. The test is described in the ASTM book [36] as a “rapid assessment of consistency and flow as well as the uniformity and the change with time of freshly mixed concrete.”

**Fig. 11:**
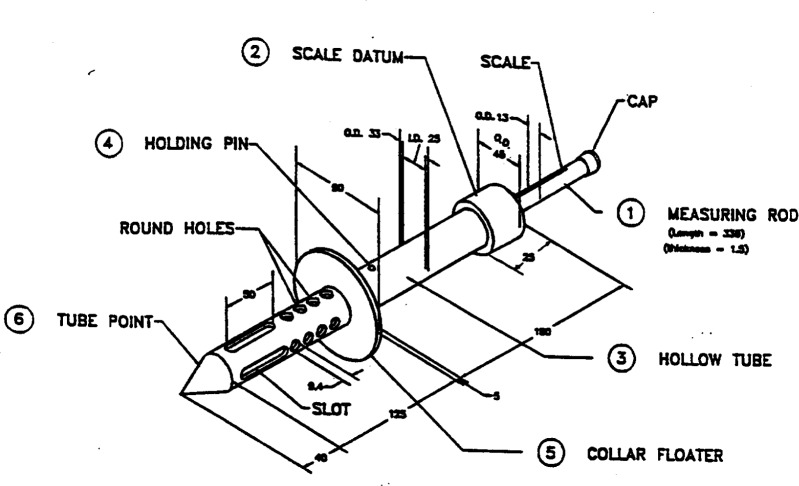
Schematic design of the probe for the flow test. (Reprinted with permission from the Annual Book of ASTM Standards, copyright American Society for Testing and Materials, 100 Barr Harbor Drive, West Conshohocken, PA 19428.)

The schematic design of the probe is shown in Fig. 11. The probe is inserted in the concrete to be tested so that the collar floater is on the concrete surface. A portion of the concrete flows into the hollow center of the probe through the perforated exterior tube. A floater or measuring rod, placed inside the perforated tube, measures how much concrete was able to flow into the probe. A higher volume corresponds to a higher ease of placement of the concrete. Nasser et al. [35] assume that the concrete flows freely into the inner tube. In reference of the two fundamental parameters (yield stress and viscosity) characterizing the rheology of concrete, this test will give a value related to the yield stress of the concrete, because the concrete will not move into the probe unless the yield stress is overcome. The stress is applied by the weight of the surrounding material. This test is suitable only for a material with low yield stress because the probe is not inserted very deeply in the concrete; therefore the stress applied by the material around the probe is not very high. It should be considered that a concrete with coarse aggregates larger than the slots in the external tube, i.e., 9.4 mm (3/8 in) in diameter, will not flow into the tube. In this case, only the mortar will flow in the device and the device will only measure the ability of the concrete to segregate.

Nasser et al. [35] showed that the values obtained with this test correlate with slump test results, although the scatter of the data is relatively high. The standard deviation is ±8 % for a single operator using the same device [36].

#### 3.1.5 Ve-be Time And Remolding Tests (Powers Apparatus)

**Fig 12:**
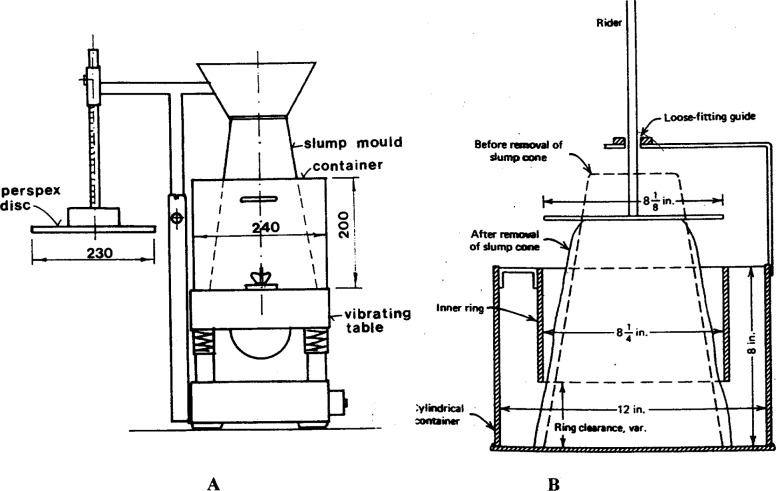
Remolding tests: A) Ve-Be test [1] (dimensions in mm), B) Powers apparatus (extracted from BS 1881: Part 103:1983 are reproduced with the permission of BSI under license *#*PD\1998 1804^2^).

These tests measure the capability of the concrete to change shape under vibration [38]. In both tests, concrete is placed in an open-ended truncated cone (Fig. 12). The time it takes the concrete to remold itself into a cylinder under vibration, after the cone is lifted away, is the output of these tests. Due to the vibrations, the concrete starts flowing after the yield stress has been overcome. Therefore, these tests can be assumed to be related to the plastic viscosity. Nevertheless, the relationship is not direct. The advantage of remolding tests is that they simulate placement of concrete under vibration, related to the field usage of concrete.

#### 3.1.6 LCL Apparatus

**Fig. 13:**
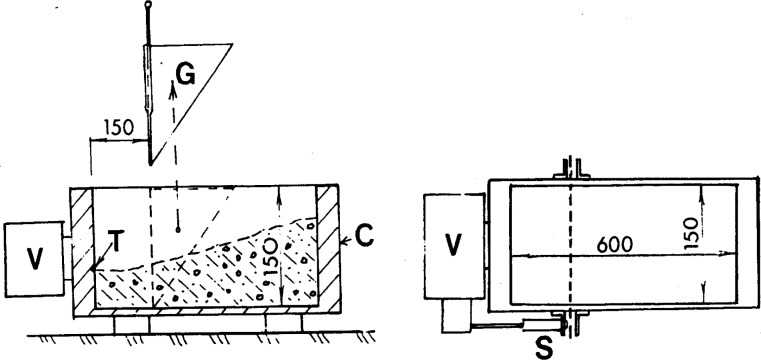
Schematic diagram of the LCL apparatus (units in mm).

The LCL [37] apparatus was developed in France, and like the remolding test, determines the time it takes for concrete to flow into a new form (Fig. 13). The main difference from the previous two tests is the geometry. The concrete is poured into a prismatic mold behind a wedge. The wedge is removed and the mold is vibrated. The operator can change the amplitude while the vibrator used determines the frequency. The time for the concrete to flow and occupy the whole prism is considered a measure of the workability. The yield stress is likely overcome by the vibration, therefore the measurement is related to the plastic viscosity of the material. If the amplitude of the vibration is slowly raised until the concrete starts flowing, a value related to the yield stress can be obtained.

#### 3.1.7 Vibration Testing Apparatus and Settling Curve, Fritsch Test [38]

**Fig. 14:**
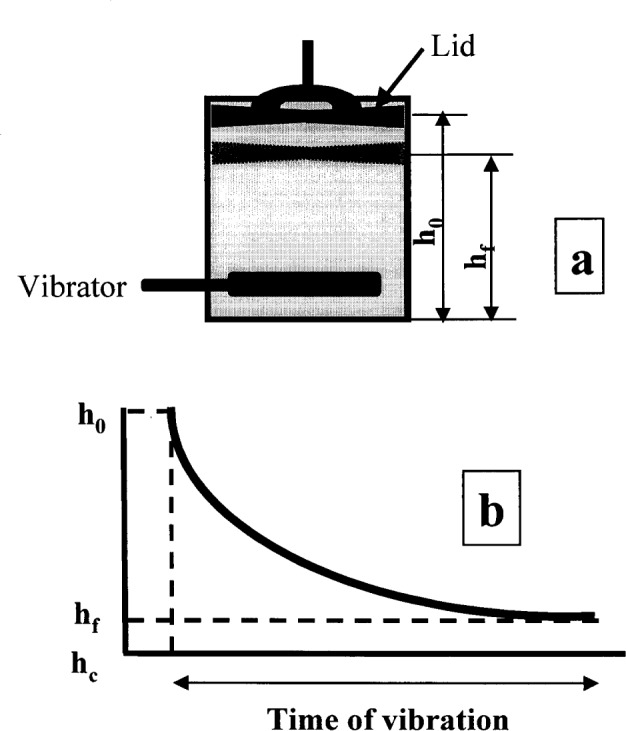
Vibration testing apparatus after Fritsch [38]; *h*_0_ is the height of the lid at the beginning of the experiment and *h*_f_ is the height at the end or at full consolidation. *h*_c_ is the asymptote for the height versus time.

The Fritsch test measures the ability of concrete to be remolded or consolidated. Figure 14a shows a schematic drawing of the apparatus. A concrete sample is placed in a container with a vibrator. The time to obtain full consolidation, i.e., time when the lid is not descending anymore, is measured. The concrete is tested under vibration, thus the shear stress is likely to be higher than the yield stress. These experimental conditions can lead to the assumption that this test will give an indication on the plastic viscosity of the concrete. But, as previously, the viscosity cannot be calculated from this value. A compaction factor can be calculated. A settling curve (Fig. 14b) is determined by plotting the height of the lid versus the time of vibration. The height after vibration, *h*_f_, is represented by the asymptote of the settling curve.

#### 3.1.8 Flow Cone

The flow cone [39] is widely used for oil well cement slurries and has been adapted for use with concrete. It consists of a funnel that is 615 mm long with a 150 mm long outlet. The upper diameter is 230 mm and the orifice diameter is 75 mm. The slope of the funnel is wall is 6:1. The amount of concrete needed is 10 L and the maximum aggregate diameter is 20 mm. The time for a given volume of concrete to pass through the orifice is measured. If the concrete starts moving through the orifice, it means that the stress is higher than the yield stress, therefore, this test measures a value that is related to the viscosity. If the concrete does not move, it shows that the yield stress is greater than the weight of the volume used. An equivalent test using smaller funnels (orifice of only 5 mm) is used for cement paste as an empirical test to determine the effect of admixtures on the flow of cement pastes. Correlation of cement paste measurements with the concrete flow was attempted with no conclusive results [40].

#### 3.1.9 Filling Ability

**Fig. 15:**
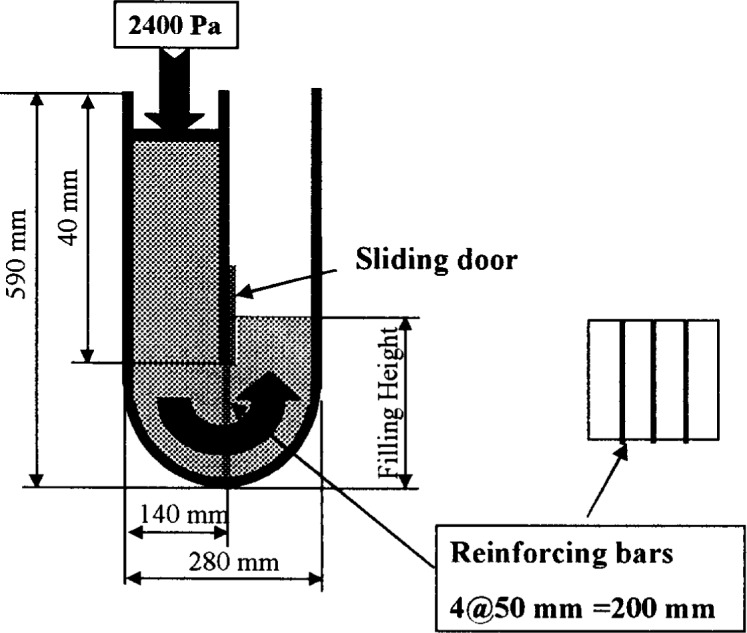
Apparatus to evaluate the filling ability of concrete under an external pressure.

**Fig. 16:**
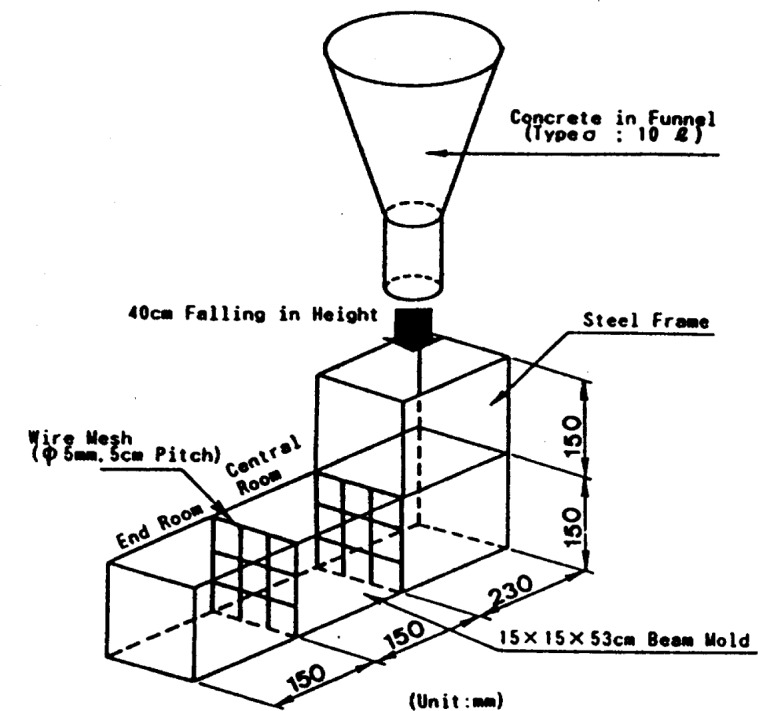
Apparatus to evaluate the filling ability of concrete under its own weight (reproduced with permission from Laboratoire Central des Ponts et Chaussees, France).

Two slightly different tests exist to measure the filling capacity of concrete, i.e., the capability of concrete to flow into a form (Figs. 15 and 16). In the first test (Fig. 15 [41]), the concrete is “pushed” through an opening partially obstructed by reinforcing bars, by applying a static pressure of about 2400 Pa. In the second test (Fig. 16 [37]), the concrete is dropped into the mold through a funnel. In both cases, the yield stress of the concrete is exceeded; therefore the value measured here is related to the viscosity. If the stress applied is lower than the yield stress, no measurement is obtained.

#### 3.1.10 Orimet Apparatus [1]

This instrument consists of a 600 mm long tube, closed at the bottom by an openable trap. The time for the concrete to flow through the long tube is recorded. The test method of the Orimet is similar to the flow cone. This test has been used for underwater concrete. The instrument is more flexible than the flow cone because the orifice or diameter of the tube can be selected to accommodate different aggregate sizes.

### 3.2 Two Factor Tests

We now examine the tests whose output gives two parameters. The values measured by these tests do not necessarily allow a direct calculation of the viscosity and yield stress. The factors measured are often indirectly related to the two fundamental parameters in a nontrivial way. The difficulty in designing correct rheological tests, tests that allow direct measurement of the fundamental parameters, is largely due to the size of the coarse aggregates, the tendency of segregation, and to the time effects. The most common fluid rheometer geometry is coaxial cylinders. In this geometry, having aggregates with a size of 10 mm or greater would force the dimensions of the instrument to be huge because these dimensions are dictated by the desirability of having a linear flow gradient between the shearing surfaces. A good approximation of this linear flow gradient can be achieved if the difference between the inner and outer radii is at least five times the diameter of the maximum size aggregate and if the ratio between the radii is held between 1 and 1.10. Therefore, the minimal dimensions, with a maximum aggregate size of 10 mm, will be 0.5 m for the radius of the inner cylinder and 0.55 m for the outer cylinder radius. These are relatively large dimensions for a relatively small maximum size aggregate.

#### 3.2.1 Tattersal Two-Point Test [4]

**Fig. 17:**
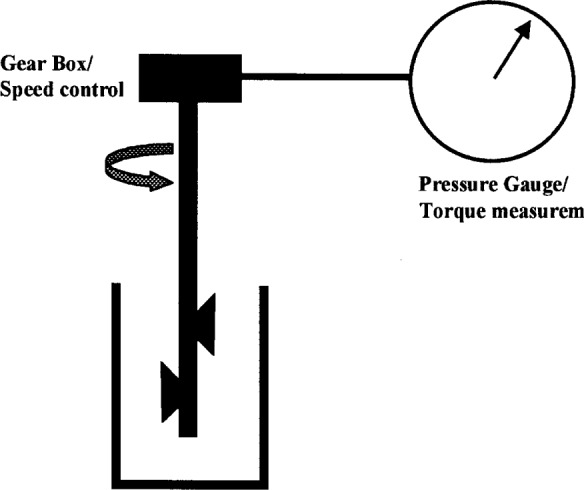
Schematic representation of Tattersall two-point rheometer.

This is the first and most widely known instrument for measuring the flow properties of concrete. The apparatus (Fig. 17) consists of a bucket containing the concrete to be tested. A vane of special geometry, or impeller, is lowered into the sample. The impeller starts rotating and the resistance on the impeller due to the material, i.e. torque, is measured. As the speed of rotation of the impeller is increased a curve of the torque versus the speed is recorded. The graph obtained is linear, therefore the stress is extrapolated to the torque at zero speed to give the yield stress and the plastic viscosity is related to the slope of the curve.

**Fig. 18:**
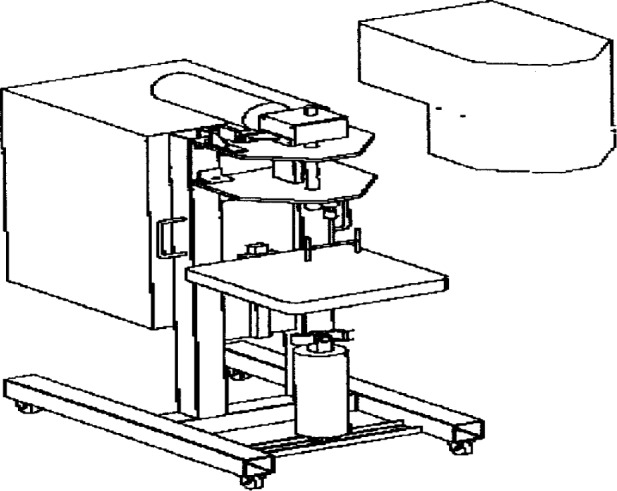
IBB Concrete Rheometer [46].

Tattersall [4] designed the first instrument, but others, Gjorv [42], Wallevick [43] and Beaupré [44,46] have improved and commercialized it. The main improvement was to automate the instrument. The torque and the speed are automatically recorded using a computer. The instrument is now available as the BML viscometer [42] or the IBB Concrete Rheometer [46] (Fig. 18). The impeller shape is not always the same, i.e., BML has a type of serrated cylinder while IBB uses an “H”-shaped impeller. The impeller of the IBB rheometer has a planetary motion in addition to an axial rotation. In both cases, the plot of torque measured versus the speed of the rotor is recorded and results in a linear relationship. The slope, *h*, and the intercept at zero speed, *g*, are related to the plastic viscosity and the yield stress, respectively. Assuming that the effective average shear rate is proportional to the speed of the impeller, Tattersall [4] gave the following equation:

$$T=(G/K)\tau_{0}+(G\eta )N$$ (2)

where:

*T* = torque
*G* = constant obtained by calibration with Newtonian fluids
*K* = constant obtained by calibration with non-Newtonian fluids
*N* = speed of the impeller
*τ*_0_ = yield stress
*η* = viscosity

Therefore, *τ*_0_ = *g*/(*G*/*K*) and *η* = *h*/*G*, where *g* and *h* are the two values measured. Unfortunately, the entities *G* and *K* are almost impossible to obtain for three main reasons:

- The flow pattern in the instrument is too complicated and not linear (or turbulent flow) to allow a calculation of *G* and *K*.
- The specimen container is too large to be able to use a standard oil to calibrate the rheometer (although some tentative calibrations were made by Tattersall [45]). We believe that the impellers will not be able to shear the whole volume of material when no aggregates are present, leading to nonvalid calibration.
- No standard granular material exists with which to calibrate the instrument.

#### 3.2.2 Bertta Apparatus [47]

This test apparatus was developed at the Technical Research Centre of Finland. Concrete is placed between two concentric cylinders of 480 mm and 330 mm diameters. The outer cylinder rotates in a oscillatory mode. The operator selects the frequency and amplitude. The torque induced by the movement is measured in the inner cylinder. This configuration allows the operator to calculate the viscosity and the yield stress of the concrete as a function of frequency. The advantage of this instrument is that is allows the operator to calculate the intrinsic rheological parameters of the materials and not only two related values, such as *g* and *h* (Tattersall device). Two issues that remain are:

- The maximum aggregate size should be limited to 13 mm (0.5 in), calculated as being 1/5 of the gap between the cylinders.
- The ratio between the radii of the two cylinders radii is 1.45. This is considered too high to have a linear flow gradient, raising the question as to whether the calculation of the rheological parameters is correct.

This instrument is not commercially available.

#### 3.2.3 The BTRHEOM Rheometer

**Fig. 19:**
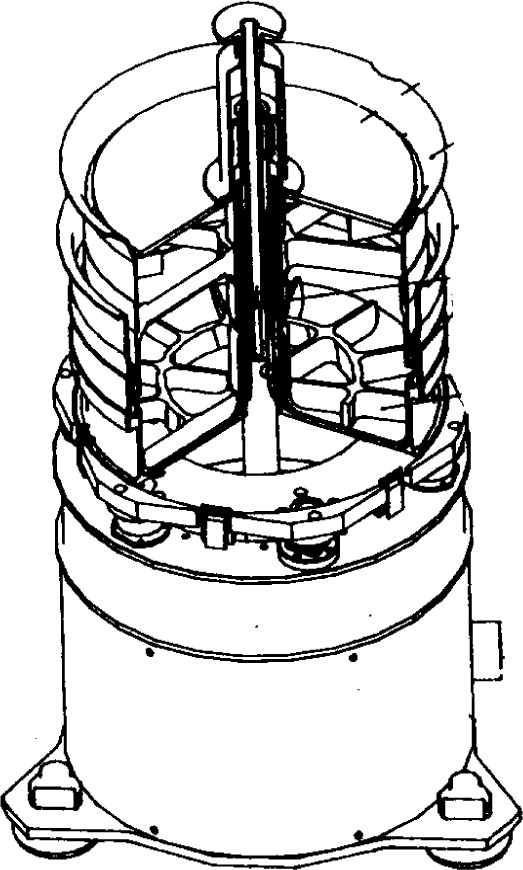
BTRHEOM instrument (reproduction with permission from LCPC).

**Fig. 20:**
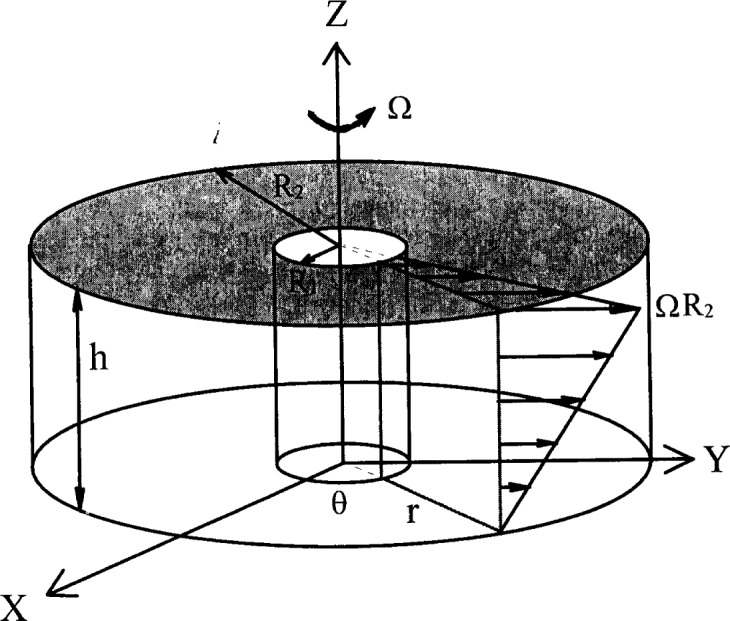
Geometry of shearing of BTRHEOM instrument [48] (reproduction with permission from LCPC).

The rheometer, BTRHEOM, was developed at the Laboratoire Central des Ponts et Chaussées (LCPC), France by de Larrard et al. [48]. It consists of a bucket with a serrated bottom, and a rotating top wheel (Fig. 19) resting on the concrete. The shear stress distribution (Fig. 20) allows direct calculation of the viscosity and yield stress according to the following equations based on the assumption that the concrete is a Bingham fluid. If this assumption is not correct, another set of equations will need to be established using the correct relationship between the shear rate and shear stress. Due to the linear pattern of flow gradient, a shear rate and shear stress can always be calculated analytically in this geometry.

$$\begin{array}{l} \tau_{0}=\frac{3\Gamma_{0}}{2\pi ({R^{3}}_{2}-{R^{3}}_{1})}\\ \eta =\frac{2h(\partial \Gamma /\partial \Omega )}{\pi ({R^{4}}_{2}-{R^{4}}_{1})} \end{array}$$ (3)

where:

*τ*_0_ = shear yield stress
*η* = viscosity
*R*_1_ and *R*_2_ = inside and outside radii of the apparatus
*h* = height of the sheared part of the sample
*Γ* = torque applied to the sample
*Ω* = angular velocity of the rotating part
*Γ*_0_ and *∂Γ*/*∂Ω* = ordinate at origin and slope of the experimental straight line *Γ*(*Ω*).

The instrument was used to collect data on shear stress versus shear rate. The results confirmed that the assumption of concrete being a Bingham fluid is correct if it had certain characteristics—These being a relatively fluid or soft concrete (typically a slump higher than 80 mm) with shear rates ranging between 0.5 s^−1^ and 8 s^−1^. De Larrard et al. [10] found that, over a wider range of shear rates, concrete behaves more like a coarse granular suspension following the Herschel-Bulkley equation (see Table 2). This apparatus permits measurements to be done under vibration. Therefore, the yield stress and the viscosity of the material can be obtained under a variety of situations.

The limitations of this instrument are the range of plastic viscosity and yield stress that can be attained, i.e., high yield stress or high plastic viscosity concretes cannot be sheared. There is always a possibility of segregation during the test especially under vibration.

#### 3.2.4 Modified Slump Cone Test [25,29]

**Fig. 21:**
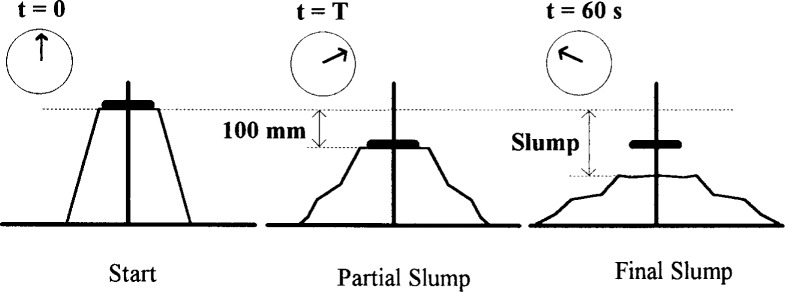
Schematics of the modified slump cone test. *T* is the “slump time” [25].

**Fig. 22:**
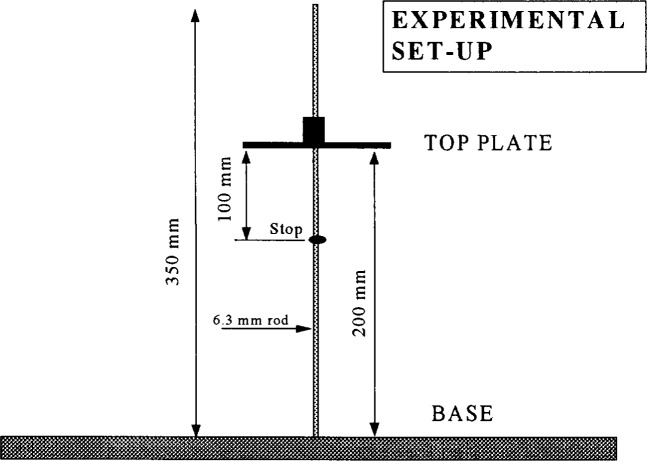
Modified slump cone device [25].

Recently, a modification of the slump cone was developed to allow the measurement of viscosity. As mentioned in Sec. 3.1.1, the standard slump test can only be correlated with the yield stress. The modification consists in measuring not only the final slump height but also the speed at which the concrete slumped. There are two methods to measure the speed at which the concrete slumped (Fig. 21):

- The original method consists of measuring the time for a plate resting on the top of the concrete to slide down with the concrete (Fig. 22) a distance of 100 mm
- Researchers at Sherbrooke University [49] eliminated the plate and shortened the central rod so that its top was 100 mm below the full slump cone height. Then the test consisted of measuring the time for the concrete to slump to the height where the rod becomes visible.

The second method has the advantage that there is no risk of the plate getting stuck, but has the disadvantage that it may be difficult to see the appearance of the rod.

**Fig. 23:**
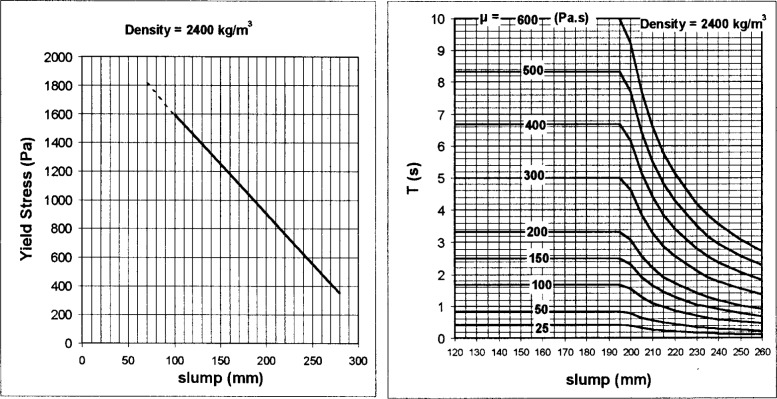
Nomographs for estimating the yield stress and plastic viscosity of concrete from the results of the modified slump test (for a concrete with a density of 2.400 kg/m^3^) [25].

The yield stress, *τ*_0_, can be calculated from the final slump, using the following empirical equation:

$$\tau_{0}=\frac{\rho }{347}(300-S)+212$$ (4)

where *ρ* is the density expressed in kg/m^3^, and *S* is the final slump in mm. The viscosity can be determined from the 100 mm slump time using an empirical equation that was determined by de Larrard et al. [25,30]. The equation used is:

$$\begin{array}{ll} \mu =\rho T\cdot 1.08\times 10^{-3}(S-175)\; & \text{for}\;200\text{mm}<S<260\text{mm}\\ \mu =25\times 10^{-3}\rho T & \text{for}\;S<200\text{mm} \end{array}$$ (5)

where *μ* is the viscosity in Pa·s and *T* is the slumping time in seconds. To facilitate the interpretation of the results, these equations can be represented in a graphic form as shown in Fig. 23.

## 4. Conclusions

Concrete flow properties need to be characterized by more than one parameter because concrete is a non-Newtonian fluid. The most commonly-used model is the Bingham equation that requires two parameters, i.e., yield stress and the plastic viscosity. The yield stress determines the stress above which the material becomes a fluid. The plastic viscosity is a measure of how easily the material will flow, once the yield stress is overcome. Other models for special concretes, such as self-leveling or self-compacting, require a more complex model, such as the Herschley-Bulkley equation.

Most of the common tests for measuring the flow properties of concrete yield only one parameter that is related either to plastic viscosity or to yield stress, or to some ill-defined combination of both. Most of these tests try to simulate field conditions and the results cannot easily be related to fundamental rheological properties. Their usage should be limited to quality control, or to check that mixture proportions have not been changed between batches. Of course, combining two tests, one related to yield stress and one related to viscosity, should give a better description of the concrete flow.

Recently, tentative steps have been made to develop tests that can measure both parameters, possibly in fundamental units. These tests are the rheometers (BTRHEOM, IBB, BML) that allow shearing at various rates, or the modified slump cone test.

This paper review has pointed out that most of the available tests are empirical. This is not a satisfactory situation for two reasons:

- It is hard, if not impossible, to relate results obtained with different tests
- The factors measured are not linked to independently measurable factors, that can be defined in fundamental physical units.

The author would like to emphasize that more research, novel tests, and models should be developed to better characterize the rheology of concrete in general and HPC in particular.

## Figures and Tables

**Table 1:** Equations relating viscosity to concentration of suspension

Equation name	Equation	Hypothesis
Einstein [6]	$\eta =\eta_{0}(1+[\eta ]\phi )$	No particle interaction, dilute suspension
Roscoe [6]	$\eta =\eta_{0}{\left(1-1.35\phi \right)}^{-K}$	Considers particle interaction
Krieger-Dougherty [5]	$\frac{\eta }{\eta_{0}}=\left(1-\frac{\phi }{\phi_{\max}}\right)-[\eta ]\phi_{\max}$	Relation between viscosity and particle packing. Takes into account the maximum packing factor
Mooney [6]	$\eta =\eta_{0}\exp\left(\frac{[\eta ]\phi }{1-\frac{\phi }{\phi_{\max}}}\right)$	Takes into account the maximum packing factor
Variable definitions *K* = constant		
*η* = Viscosity of the suspension *η*_0_ = Viscosity of the liquid/media		
*ϕ* = Volume fraction of solid [*η*] = Intrinsic viscosity of the suspension, (2.5 for spheres)		
*ϕ*_max_ = Maximum packing factor		

**Table 2:** Equations relating shear stress and shear rate

Equation name	Equation
Newtonian [1]	$\tau =\eta \dot{\gamma }$
Bingham [4]	$\tau =\tau_{0}+\eta \dot{\gamma }$
Herschel and Bulkley [7]	$\tau =\tau_{0}+K{\dot{\gamma }}^{n}$
Power equation [7]	$\tau =A{\dot{\gamma }}^{n}$
	*n* = 1 Newtonian flow
	*n* > 1 shear thickening
	*n* < 1 shear thinning
Vom Berg [8], Ostwald-deWaele [4]	$\tau =\tau_{0}+B\sinh^{-1}(\dot{\gamma }/C)$
Eyring [7]	$\tau =a\dot{\gamma }+B\sinh^{-1}(\dot{\gamma }/C)$
Robertson-Stiff [7]	$\tau =a{\left(\dot{\gamma }+C\right)}^{b}$
Atzeni et al. [9]	$\dot{\gamma }=a\tau^{2}+\beta \tau +\delta$
Variable definitions	
*τ* = Shear stress	*η* = Viscosity
*τ*_0_ = Yield stress	$\dot{\gamma }=\text{Shear rate}$
*A*,*a*,*B*,*b*,*C*,*K*, *α*, *β*, *δ* = constants	

**Table 3:** Examples of definition of workability by various societies [14]

Name of society	Definition
American Concrete Institute (ACI)	That property of freshly mixed concrete or mortar which determines the ease and homogeneity with which it can be mixed, placed, compacted, and finished
British Standards Institution	That property of fresh concrete, mortar, or the like, which determines the ease with which it can be manipulated and fully compacted
Association of Concrete Engineers, Japan	That property of freshly mixed concrete or mortar which determines the ease with which it can be mixed, placed and compacted due to its consistency, the homogeneity with which it can be made into concrete, and the degree with which it can resist separation of materials

## Footnotes

1 Certain commercial equipment, instruments, or materials are identified in this paper to foster understanding. Such identification does not imply recommendation or endorsement by the National Institute of Standards and Technology, nor does it imply that the materials or equipment identified are necessarily the best available for the purpose.

2 Complete editions of the standards can be obtained from BSI Customer Services, 389 Chiswick High Rd., London W4 4AL, Great Britain.
